# Hidden highways: fungi associated with glossiphoniid leeches

**DOI:** 10.1016/j.ijppaw.2025.101158

**Published:** 2025-11-10

**Authors:** Maciej Grobelski, Michał Kułakowski, Karolina Górzyńska

**Affiliations:** a Department of Animal Morphology, Faculty of Biology, Adam Mickiewicz University, Uniwersytetu Poznańskiego 6, 61-614, Poznan, Poland; b Department of Systematic and Environmental Botany, Faculty of Biology, Adam Mickiewicz University, Uniwersytetu Poznańskiego 6, 61-614, Poznan, Poland

**Keywords:** Fungal diversity, Glossiphoniidae, Leech, Mycobiome, Plant pathogens

## Abstract

Fungi associated with aquatic invertebrates remain poorly characterized, particularly those inhabiting leeches (Hirudinea), which are key components of freshwater ecosystems. In this study, the fungal communities associated with five species of glossiphoniid leeches were investigated, and isolates were obtained and compared from internal tissues and the body surface. In total, 19 fungal species were identified, including plant pathogens (e.g., *Cadophora luteo-olivacea*, *Comoclathris typhicola*, *Plectosphaerella plurivora*) and opportunistic human pathogens (e.g., *Meyerozyma guilliermondii*, *Mucor circinelloides*, *Arthroderma* sp.). Notably, *Papiliotrema aurea*, a fungus known to infect invertebrate tissues, was detected inside *Glossiphonia complanata*. The occurrence of several taxa both in the surrounding water and on leeches suggests that leeches may act as vectors of fungal dispersal in aquatic ecosystems. The results highlight the importance of leeches not only as potential dispersal agents of plant pathogens, which may affect vegetation and aquaculture, but also as reservoirs of opportunistic human pathogens.

## Highlights

• Fungal species were identified from internal and external leech tissues.
• Leeches harbor both plant and opportunistic human pathogenic fungi.
• Leeches may serve as vectors for fungal transmission in aquatic habitats.

## Graphical abstract

**Figure:**
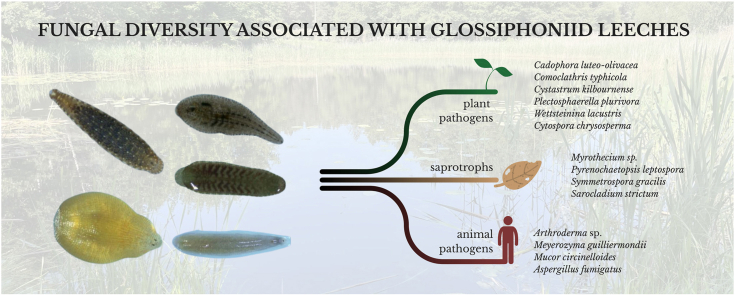

## 1 Introduction

Interactions between fungi and animals span a wide ecological spectrum and are widespread in nature. Yet, many animal-associated fungi remain poorly characterized, as they are often ephemeral, inconspicuous, and challenging to study, frequently requiring specialized methodologies. This knowledge gap is particularly pronounced for fungi associated with aquatic invertebrates, despite their potential ecological and epidemiological importance. Among these, fungi associated with leeches (Hirudinea) are especially understudied, even though leeches play a vital role in freshwater ecosystems.

Although over 700 species of leeches are currently recognized, information on their associations with fungi remains scarce. These interactions are particularly interesting in terms of leeches acting as vectors, potentially facilitating the colonization and dissemination of specific fungal species (Jacobsen et al., 2017). The best-studied group of leeches in the context of microbial vectors are medicinal leeches (*Hirudo* spp., *Macrobdella decora*), which are specialized in feeding on the blood of vertebrates and are widely used in hirudotherapy (Graf, 1999; Bauters et al., 2007; Yantis et al., 2009; Bourdais et al., 2010; Nelson and Graf, 2012; Litwinowicz and Blaszkowska, 2013). One of the most commonly found bacteria in leeches are *Aeromonas* species – *Aeromonas hydrophila*, *Aeromonas jandaei*, and *Aeromonas veronii* –which inhabit the digestive system of medicinal leeches (Graf, 1999; Bauters et al., 2007; Laufer et al., 2008; Siddall et al., 2007, 2011; Bourdais et al., 2010; Nelson and Graf, 2012). In addition, among the bacteria inhabiting leech bodies, one can find – *Serratia marcescens*, *Pseudomonas* spp., *Vibrio fluvialis* (Porshinsky et al., 2011), *Morganella morganii* (Dowd et al., 2008; Laufer et al., 2008; Whitaker et al., 2014; Rahmati Holasoo et al., 2022), and Klebsiella *pneumoniae* (Yibin et al., 2020). Leeches have been reported to harbor representatives of *Rikenella* (Bomar et al., 2011) and *Rickettsia* (Kikuchi and Fukatsu, 2005). They can also act as carriers of parasites – helminths, primarily flukes and tapeworms, serving as their intermediate hosts (Riggs and Ulmer, 1983; Siciński, 2013).

Medicinal leeches, particularly those from specialized breeding facilities, have also been reported as hosts for fungi (Litwinowicz and Blaszkowska, 2013). The most abundant group was fungi of the genus *Candida*, which can cause complications following hirudotherapy procedures (Biedunkiewicz and Bielecki, 2010; Litwinowicz and Blaszkowska, 2013). Other fungal taxa associated with medical leeches included *Lipomyces starkeyi*, *Rhodosporidium* sp., *Schizosaccharomyces* sp., *Trichosporon asahii*, *Trichosporon asteroides*, *Trichosporonoides oedocephalis*, *Yarrowia lipolytica* (Biedunkiewicz and Bielecki, 2010; Litwinowicz and Blaszkowska, 2013), *Debaryomyces* spp., *Kluyveromyces* spp., *Metschnikowia pulcherrima*, *Pichia guilliermondi* (current name: *Meyerozyma guilliermondi*), *Stephanoascus farinosus*, *Oosporidium* sp. (Biedunkiewicz and Bielecki, 2010). In addition to medicinal leeches, previous reports also concern the leech *Theromyzon maculosum* (Rathke, 1862), in which two major groups of fungi were identified: internal (digestive system and pharynx) and external (body surface). The species composition varied between these two groups: the first group included *Kluyveromyces*, *Saccharomyces*, *Citeromyces*, *Lodderomyces*, and *Debaryomyces*, while the second group comprised *Candida*, *Pichia*, and *Debaryomyces* (Biedunkiewicz et al., 2023).

The role of leeches as vectors of fungi is particularly important due to the significant ecological functions that fungi play in aquatic environments. Fungi participate in the decomposition of organic matter, humus, and chitin, the degradation of macrophytes, as well as detoxification. In addition to these beneficial effects on ecosystems, fungi can also have numerous negative impacts – primarily pathogenicity. Pathogens may attack phytoplankton, zooplankton, as well as aquatic invertebrates and vertebrates (Wong et al., 1998; Grossart et al., 2019). Fungi can also be found in drinking water sources, and contamination with pathogenic fungi may pose a risk to human health (Mhlongo et al., 2019; Babič and Gunde-Cimerman, 2023; Hussain et al., 2024).

Leeches can transmit pathogenic organisms not only through feeding, but also via contact between their bodies and an open wound, as well as during attempts to remove them (Yantis et al., 2009). It is highly likely that leeches also facilitate horizontal transmission of pathogens, particularly those located on their body surface, which can occur during copulation (Nelson and Graf, 2012). Experimental studies have shown that bacteria, viruses, and parasitic protozoa can survive in leeches for several months (Nehili et al., 1994; Al-Khleif et al., 2011).

The aim of our study is to examine and compare the species composition of fungi found inside and on the bodies of selected leech species from the family Glossiphoniidae. This family is characterized by high species diversity, as well as a variety of hosts, prey, and habitats in which leeches occur, providing an opportunity to investigate the ecological factors influencing fungal colonization of specific leech species. Gaining this knowledge will constitute a crucial first step toward understanding fungal-leech associations, potentially revealing hidden ecological networks and the subtle ways in which fungi influence aquatic biodiversity.

## 2 Materials and methods

### 2.1 Collection of leeches

Leeches were collected during two sampling periods: in July 2024 and from May to June 2025. Specimens were obtained from two aquatic sites located on the Morasko campus of Adam Mickiewicz University in Poznań: a section of the “Różany Potok” stream (52°27′52.8″N 16°55′40.2″E) and a retention reservoir (52°27′52.7″N 16°55′26.1″E). The leeches were gathered from various abiotic and biotic substrates found in and on the water, mainly branches, stones, and aquatic vegetation. Each individual was placed in a separate 15 ml Falcon tube filled with water from the site where it was collected. At the same time, water samples were taken from the reservoirs for control analysis of aquatic fungi. In total, 23 leeches belonging to the family Glossiphoniidae were collected.

The leeches were transported to the microscopy laboratory of the Department of Animal Morphology at Adam Mickiewicz University in Poznań. Live identification of leech species was carried out using an identification key to native leech species developed by Maciej Grobelski (unpublished).

### 2.2 Isolation of fungi

Surface-associated fungi from leeches were isolated following a method adapted from Ozdal et al. (2012). Individual leeches were transferred into sterile Eppendorf tubes containing autoclaved distilled water. Each tube was vortexed briefly and then left to stand for 10 min. Subsequently, a 10^−1^ dilution was prepared by transferring 100 μL of the sample into a new Eppendorf tube containing 900 μL of autoclaved distilled water, followed by vortexing. From each tube, 150 μL was plated onto PDA medium (Potato Dextrose Agar, OXOID) containing chloramphenicol (100 mg/L, A&A Biotechnology) using a sterile spreader. Chloramphenicol was added to the medium as an antibiotic to inhibit bacterial growth.

To isolate fungi from the internal tissues of leeches, each individual was euthanized in 70 % ethanol and left for 10 min to allow for surface sterilization. Then each individual was transferred into a fresh Eppendorf tube containing 1 mL of autoclaved distilled water, vortexed briefly and left to stand for an additional 10 min. Afterward, each leech was sectioned transversely into three parts, and the tissue fragments were placed onto PDA medium containing chloramphenicol. To assess the effectiveness of surface sterilization, 150 μL of the final ethanol rinse water was plated onto PDA medium using a sterile spreader.

Aquatic fungi were isolated from water samples (250 mL each) collected in triplicate from every reservoir. From each sample, tenfold serial dilutions (10^−1^ and 10^−2^) were prepared in triplicate using sterile, autoclaved distilled water. Aliquots of 150 μL from each dilution, as well as from the undiluted sample, were spread onto PDA medium containing chloramphenicol using a sterile spreader.

All procedures were performed under aseptic conditions within a laminar flow hood. Plates were incubated at 25 °C, and any emerging fungal colonies were transferred onto fresh PDA medium for further analysis.

### 2.3 Identification of fungi

Molecular identification was performed on the isolates. From each cultured fungal colony, DNA was extracted using the Quick-DNA Fungal/Bacterial Miniprep Kit (Zymo Research, USA) and stored at −20 °C. PCR amplification was performed using the ITS1F (Gardes and Bruns, 1993) and ITS4 (White et al., 1990) primers, which anneal to the ribosomal DNA cassette comprising partial SSU, ITS1, 5.8S, ITS2, and partial LSU regions. The PCR reactions were carried out in a total volume of 25 μL containing 2.5 μL of 10X buffer, 2.5 μL of 2.5 mM dNTP mix, 0.5 μL of each primer (10 μM), 0.5 μL of Taq DNA polymerase, and 50 ng of DNA template. Amplification was performed in a thermocycler under the following conditions: initial denaturation at 95 °C for 2 min; 35 cycles of denaturation at 95 °C for 30 s, annealing at 55 °C for 30 s, and extension at 72 °C for 60 s; followed by a final extension at 72 °C for 5 min. PCR products were purified enzymatically using alkaline phosphatase and exonuclease I, then directly sequenced using the ABI BigDye Terminator v3.1 Cycle Sequencing Kit (Applied Biosystems, USA). Consensus sequences were assembled using BioEdit software and subsequently submitted to GenBank. Sequence identification was performed by comparing the consensus sequences against those available in the European Molecular Biology Laboratory (EMBL) nucleotide database and the NCBI database (www.ncbi.nlm.nih.gov) using the BLAST algorithm (Altschul et al., 1990). A species-level identification was considered valid when the ITS region sequence shared ≥98 % identity with a reference sequence from the databases.

For isolates yielding ambiguous results, morphological identification was performed. The fungal morphology was studied macroscopically by observing the colony features (color, shape, size, and hyphae), and microscopically using a light microscope (Olympus BX53F) to examine conidiophores, spores, and other characteristic structures. Microscopic observations were performed on preparations made using the Slide Culture Method. Briefly, pieces of sterile filter paper were placed on the bottom of a sterile Petri dish and moistened with 1 mL of sterile water. Four cut caps from Eppendorf tubes were then positioned on the paper to serve as supports. A sterile microscope slide was placed on top of the caps, and a 1 × 1 cm block of PDA medium was placed at the center of the slide. The fungal isolate was inoculated onto the sides of the agar block using a sterile inoculating loop, and a coverslip was gently placed on top. The Petri dish was sealed with Parafilm and incubated at 25 °C for 5 days. All procedures were carried out under sterile conditions in a laminar flow cabinet. After incubation, the coverslip was carefully removed and placed on a new microscope slide containing a drop of lactophenol cotton blue for microscopic examination.

## 3 Results

**Fig. 1:**
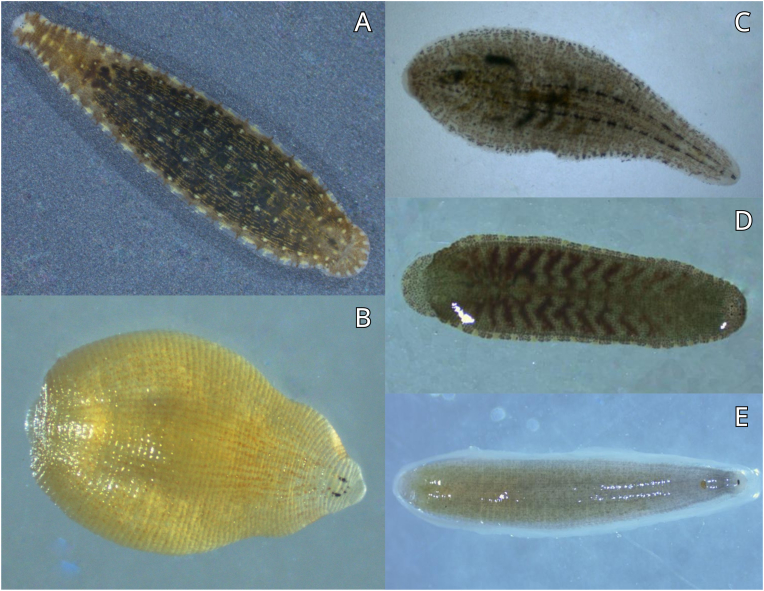
Leech species (Hirudinea: Glossiphoniidae) investigated in the study. A – *Hemiclepsis marginata*; B – *Alboglossiphonia hyalina*; C – *Glossiphonia complanata*; D – juvenile *Theromyzon tessulatum*; E − *Helobdella stagnalis.*

Among the 23 collected leeches, the following species were identified: *Alboglossiphonia hyalina* (Müller, 1774) (5 individuals), *Glossiphonia complanata* (Linnaeus, 1758) (5 individuals), *Helobdella stagnalis* (Linnaeus, 1758) (5 individuals), *Hemiclepsis marginata* (Müller, 1773) (5 individuals), and *Theromyzon tessulatum* (Müller, 1774) (3 individuals) (Fig. 1).

**Fig. 2:**
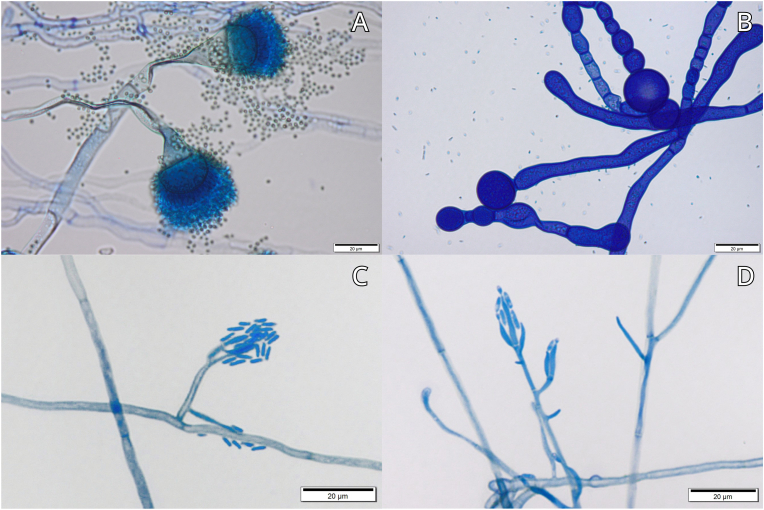
Selected fungal taxa isolated from leeches (Hirudinea: Glossiphoniidae) observed under a light microscope. A – conidiophores and conidial spores of *Aspergillus fumigatus*; B – chlamydospores of *Mucor circinelloides*; C–D – conidiophores of *Cadophora luteo-olivacea*.

A total of 19 fungal taxa were isolated from five leech species, with clear differences in richness both among hosts and between internal and external sources (Table 1, Fig. 2). Across all hosts, external surfaces yielded a higher number of taxa (15) compared to the internal environment (6). *Comoclathris typhicola* was among the most frequently isolated species, occurring in three leech hosts (*Alboglossiphonia hyalina*, *Hemiclepsis marginata*, and *Theromyzon tessulatum*). Similarly, *Aspergillus fumigatus* was detected in three species (*A. hyalina*, *Glossiphonia complanata*, and *Helobdella stagnalis*). Notably, *A*. *fumigatus* was the only taxon present both internally and externally, a pattern observed exclusively in *A. hyalina*.

**Table 1:** Leech species (Hirudinea: Glossiphoniidae) and fungal taxa isolated from internal tissues and body surfaces. Taxa marked with an asterisk (∗) were also recorded in water samples.

Leech species	Internal fungi	External fungi
*Alboglossiphonia hyalina*	*Aspergillus fumigatus*	*Aspergillus fumigatus*
		*Comoclathris typhicola∗*
*Glossiphonia complanata*	*Arthroderma* sp.	*Aspergillus fumigatus*
	*Cladosporium* sp.	*Cladosporium* sp.
	*Papiliotrema aurea*	*Diatrype stigma*
	*Plectosphaerella plurivora*	*Meyerozyma guilliermondii*
	*Pseudoscopulariopsis* sp.	*Mucor circinelloides∗*
		*Pyrenochaetopsis leptospora∗*
		*Wickerhamomyces onychis*
*Helobdella stagnalis*	*Papiliotrema* sp.	*Aspergillus fumigatus*
		*Cadophora luteo-olivacea*
		*Cladosporium* sp.
		*Syncephalastrum* sp.
*Hemiclepsis marginata*		*Comoclathris typhicola∗*
		*Cystastrum kilbournense*
		*Meyerozyma guilliermondii*
		*Myrothecium* sp.
		*Sarocladium strictum∗*
		*Wettsteinina lacustris∗*
*Theromyzon tessulatum*		*Comoclathris typhicola∗*
		*Symmetrospora gracilis*

The greatest fungal richness was recorded in *Glossiphonia complanata* (11 taxa in total), comprising five internal and six external isolates. The lowest diversity was observed in *Alboglossiphonia hyalina*, where only *Aspergillus fumigatus* was found both internally and externally, with an additional external occurrence of *Comoclathris typhicola*. Two leech species, *Hemiclepsis marginata* and *Theromyzon tessulatum*, were characterized exclusively by external fungal isolates.

Fungi were isolated from water samples collected from tanks where leeches were obtained. The isolates included *Comoclathris typhicola*, *Cytospora chrysosperma*, *Didymella* sp., *Mucor circinelloides*, *Penicillium* sp., *Pyrenochaetopsis leptospora*, *Sarocladium strictum*, *Trichoderma* sp., and *Wickerhamomyces onychis*. Among these, *Comoclathris typhicola* and *Wickerhamomyces onychis* were the most prevalent in the water. Interestingly, five species: *Comoclathris typhicola*, *Mucor circinelloides*, *Pyrenochaetopsis leptospora*, *Sarocladium strictum*, and *Wickerhamomyces onychis* were detected both in the water and on leech body surfaces.

## 4 Discussion

In this study, we examined the presence and species composition of fungi found both inside and on the bodies of selected leech species from the family Glossiphoniidae. We identified 19 fungal taxa, representing 19 genera belonging to 18 families, with the majority occurring on the body surface. Isolated fungi represent a wide range of ecological groups, including plant pathogens, opportunistic human pathogens, and saprotrophs.

Plant-pathogenic taxa included *Cadophora luteo-olivacea,* which has been previously recovered from apple, pear, grapevine, plum, and blueberry, and is also responsible for apple side rot (Abreo et al., 2012; Amaral Carneiro et al., 2022), *Comoclathris typhicola* has been reported from cattail (*Typha latifolia*) (Ahmadpour et al., 2024), *Cystastrum kilbournense* from maize leaves (Kurtzman and Robnett, 2015) and *Plectosphaerella plurivora* from false ginseng (*Panax notoginseng*) (Han et al., 2020). Likewise, *Wettsteinina lacustris* is associated with narrowleaf cattail (*Typha angustifolia*) (Aptroot, 1998), common club-rush (*Schoenoplectus lacustris*), and cattail (*Typha latifolia*) (Müller, 1950). Because the life cycle of leeches is closely linked to aquatic plants that provide them with shelter (Pawłowski, 1936; Elliott and Dobson, 2015), the presence of these fungi on leeches may indicate ecological interactions between leeches and aquatic vegetation. It is therefore possible that leeches play a role in the passive transfer of plant-pathogenic fungi in freshwater ecosystems, although further studies are required to confirm this mechanism.

Although the majority of isolates were saprotrophic fungi, typically associated with decaying organic matter and cellulose degradation, such as *Myrothecium* sp. (Sarbhoy and Kulshreshtha, 1999), human-associated pathogens were also detected among the isolates. These included: (1) *Arthroderma* sp., with certain species and strains known to cause dermatophytosis (Hainsworth et al., 2021); (2) *Meyerozyma guilliermondii*, an opportunistic yeast responsible for invasive infections and candidemia in immunocompromised patients (Ghasemi et al., 2022); (3) *Mucor circinelloides*, a well-known agent of mucormycosis (Khan et al., 2009); and (4) members of the genus *Syncephalastrum*, some of which have been implicated in fungal sinusitis (Almenara et al., 2024). It is worth noting that some leech species included in our study, such as *Hemiclepsis marginata* (a parasite of fish) and *Theromyzon tessulatum* (a parasite of waterfowl), interact with vertebrate hosts. Although no cases of fungal transmission by these species have been reported to date, their host associations justify further investigation of whether fungi associated with leeches could occasionally come into contact with susceptible wildlife.

The association of fungi with leeches may potentially influence their dispersal, as leeches could serve as incidental carriers of these organisms. This is particularly relevant in the case of fungi detected in the leech *Theromyzon tessulatum*. This leech species is characterized by its wide geographic distribution – the Americas, Eurasia, and Africa – and its ability to be transported during bird migrations (Mabrouki et al., 2025), which could indirectly facilitate the movement of associated microorganisms. A remarkable feature of this leech is its parasitism on the mucous membranes of the nasal cavities and eyes of birds, mainly members of the Anatidae family, but also grebes and domestic poultry (Pawłowski, 1936; Auw-Haedrich et al., 1998; Elliott and Dobson, 2015). Such host interactions may offer occasional opportunities for fungi to come into contact with vertebrates, although no evidence currently confirms active transmission. The literature describes a single case of *T. tessulatum* occurrence in the human eye, suggesting that although this species typically parasitizes birds, it may occasionally parasitize atypical hosts. However, the epidemiological significance of such cases is considered very low and likely represents accidental events rather than a genuine threat to public health (Auw-Haedrich et al., 1998).

Other leech species exhibit lower dispersal abilities and prey mainly on invertebrates (Pawłowski, 1936; Wrona et al., 1979, 1981; Elliott and Dobson, 2015). This is particularly important for the fungus *Papiliotrema aurea*, which in our study was isolated from the leech *Glossiphonia complanata*. Previously, this fungus was reported from the muscles and hepatopancreas of the Chinese mitten crab (*Eriocheir sinensis*) (Liu et al., 2022), suggesting that it may be a generalist species capable of colonizing diverse aquatic organisms. Its occurrence in leeches may therefore reflect environmental exposure or ingestion rather than a specific host relationship. While the possibility of leeches acting as passive carriers of aquatic microorganisms cannot be excluded, additional studies would be required to verify any active role in fungal dispersal.

It is also worth noting that the composition of the leech mycobiome may vary depending on environmental factors such as eutrophication or anthropogenic pollution, which could shape the diversity and distribution of associated fungi. Even under extreme Antarctic conditions, marine animals form rich and often unexpected associations with fungi. When compared with our observations in leeches, these findings emphasize that animal-fungus interactions are widespread and may play an important ecological and evolutionary role (Godinho et al., 2019).

Although some of the fungi detected in this study have pathogenic potential, there is currently no evidence that leeches actively transmit these organisms to wildlife or humans. The possible role of leeches as reservoirs or vectors remains hypothetical and requires targeted studies, including controlled transmission experiments, molecular comparisons of fungal strains, and broader sampling across multiple leech species and habitats. It is also important to emphasize the need for further research to determine whether leeches may act as reservoirs and/or vectors in the transmission of fungi. Future studies should include broader and more systematic sampling across different freshwater habitats, combined with metagenomic approaches that allow comparisons between the mycobiota of leeches, their environment, and potential hosts. Basic molecular analyses and histological examinations could help clarify whether the detected fungi are merely surface-associated or can be transferred during feeding. Expanding the research to include a larger number of leech species and increasing sample sizes will be essential to assess whether potential fungal transmission is species-specific or more widespread.

In summary, the diversity of fungal species isolated from leeches highlights not only the potential role of leeches as vectors but also their ecological importance in the cycling of organic matter within aquatic environments. Although some detected species are known pathogens, the available evidence is not sufficient to confirm that leeches actively contribute to the spread of infections in aquaculture. Nevertheless, the presence of pathogenic fungi indicates that leeches may play a role in their passive dissemination, which, in a broader perspective, could have significant ecological and economic implications (Paul et al., 2018; Deng et al., 2025). Furthermore, the detection of species potentially pathogenic to humans (e.g., *Mucor circinelloides* or *Meyerozyma guilliermondii)* suggests possible epidemiological relevance of these organisms, particularly in the context of human contact with natural waters and leeches (Cichocka et al., 2021). Future research should focus on metagenomic analyses and comparative studies of the fungal microbiome across different leech species and their habitats, as well as experimental approaches to determine whether these fungi actively colonize leech tissues or are passively transported, providing a deeper understanding of the scale and mechanisms underlying these interactions.

## CRediT authorship contribution statement

**Maciej Grobelski:** Writing – review & editing, Writing – original draft, Visualization, Methodology, Investigation, Formal analysis, Data curation, Conceptualization. **Michał Kułakowski:** Writing – review & editing, Writing – original draft, Visualization, Project administration, Methodology, Investigation, Funding acquisition, Formal analysis, Data curation, Conceptualization. **Karolina Górzyńska:** Writing – review & editing, Supervision, Conceptualization.

## Conflict of interest

The authors declare that they have no known competing financial interests or personal relationships that could have appeared to influence the work reported in this paper.
